# Dental Findings in Patients With Non-surgical Hypoparathyroidism and Pseudohypoparathyroidism: A Systematic Review

**DOI:** 10.3389/fphys.2018.00701

**Published:** 2018-06-19

**Authors:** Jane Hejlesen, Line Underbjerg, Hans Gjørup, Agnes Bloch-Zupan, Tanja Sikjaer, Lars Rejnmark, Dorte Haubek

**Affiliations:** ^1^Section for Pediatric Dentistry, Department of Dentistry and Oral Health, Health, Aarhus University, Aarhus, Denmark; ^2^Department of Endocrinology and Internal Medicine, Aarhus University Hospital, Aarhus, Denmark; ^3^Center for Oral Health in Rare Diseases, Department of Maxillofacial Surgery, Aarhus University Hospital, Aarhus, Denmark; ^4^Faculté de Chirurgie Dentaire, Institut d'Etudes Avancées, USIAS, FMTS, RARENET Interreg V, Université de Strasbourg, Strasbourg, France; ^5^Pôle de Médecine et Chirurgie Bucco-Dentaires, Centre de Référence des Maladies Rares Orales et Dentaires, O-Rares, Hôpitaux Universitaires de Strasbourg, Strasbourg, France; ^6^Institut de Génétique et de Biologie Moléculaire and Cellulaire, Centre Européen de Recherche en Biologie et en Médecine, Université de Strasbourg, Centre National de la Recherche Scientifique UMR7104, Institut National de la Santé et de la Recherche Médicale U964, Illkirch, France

**Keywords:** hypoparathyroidism, pseudohypoparathyroidism, 22q11 deletion syndrome, dental anomalies, enamel hypoplasia

## Abstract

**Background:** Dental aberrations have been mentioned in relation to non-surgical hypoparathyroidism (Ns-HypoPT) and pseudohypoparathyroidism (PHP). However, a systematic review of dental characteristics have not been performed. The present systematic review describes the dental findings in patients with Ns-HypoPT and PHP.

**Methods:** Studies on Ns-HypoPT and PHP reporting dental features were eligible. A systematic literature search was conducted using four bibliographic databases (Web of Science, Scopus, Pubmed, and Embase) and was limited to studies written in English. Reviews, meta-analyses and letters were excluded. Both the research and reporting of results were based on PRISMA (preferred Reporting Items for Systematic Reviews and Meta-Analysis) guidelines.

**Results:** Of 88 studies included, nine were cross-sectional, one was a prospective cohort study, 26 were case series, and 52 were case reports. The most frequently reported findings in patients with Ns-HypoPT were enamel opacities, enamel hypoplasia, hypodontia, and eruption disturbances. In patients with PHP, enamel hypoplasia, eruption disturbance, and deviation of the root morphology were the most frequently reported findings.

**Conclusion:** An association between enamel hypoplasia and Ns-HypoPT and PHP is likely. The results should, however, be interpreted cautiously due to the limited number of high-quality studies. The present review confirms the need of further well-designed studies, such as large-scale studies, e.g., multicenter studies, to conclude on the reported associations between Ns-HypoPT/PHP and enamel hypoplasia.

## Introduction

Hypoparathyroidism (HypoPT) is characterized by low levels of plasma calcium with inappropriately low levels of parathyroid hormone, as well as high phosphate levels. The most common cause of HypoPT is following neck surgery (Marx, 2000). Non-surgical hypoparathyroidism (Ns-HypoPT) can be induced by various etiologies, either by genetic mutations, autoimmune diseases, radiation, sarcoidosis, or accumulation of iron or cobber. The genetic types are dominated by 22q11 deletion syndrome (22q11DS) and the autoimmune diseases are dominated by autoimmune polyglandular syndrome type 1 (APS 1). The most common genetic reasons for Ns-HypoPT is 22q11 deletion, resulting in hypoplasia of the parathyroid glands. The scientific literature reports that up to 20% of all patients with 22q11 deletion develop chronic HypoPT, and up to 60% of all patients with 22q11 deletion have latent HypoPT (Olesen et al., 2010). Previously, 22q11DS was known as DiGeorge syndrome (OMIM#188400) or Velocardiofacial syndrome (OMIM#192430). Another well-known source of hypocalcemia with low PTH levels is autosomal dominant hypocalcemia (ADH) triggered by an activating mutation in the genes encoding the calcium-sensing receptor (*CaSR*) mainly placed in the parathyroid glands (Bilezikian et al., 2011). Autoimmune HypoPT is caused by mutations in *AIRE*, resulting in APS 1 (Bilezikian et al., 2011). The classification and diagnostic criteria of genetic and autoimmune causes of Ns-HypoPT is, however, a challenge. In the literature, idiopathic hypoparathyroidism (IHP) designates HypoPT with unknown etiology.

Pseudohypoparathyroidism (PHP) is characterized by end-organ resistance to PTH. PHP is caused by mutations in either *GNAS, STX16*, or *GNASAS1* on the maternal allele of chromosome 20q13 and is subdivided into groups depending on the clinical and hormonal phenotypes: type 1a (known as Albright Hereditary Osteodystrophy, AHO; OMIM #103580), type 1b (OMIM #603233), type 1c (OMIM#612464), and type 2 (OMIM #203390). Patients with PHP may also have symptoms related to insufficiency of other G-protein coupled hormones, especially TSH. Despite the general peripheral resistance to PTH, patients with PHP may have organ specific variations in their sensitivity to circulating PTH. This is believed to be attributable to genetic imprinting. AHO without biochemical abnormalities is known as pseudopseudohypoparathyroidism (PPHP) and is due to mutation of *GNAS* on the paternal allele of chromosome 20q13 (OMIM#612463).

A number of dental findings have been reported in relation to the diseases mentioned above (Illum et al., 1981; Jensen et al., 1981). Low levels of PTH in Ns-HypoPT and end-organ resistance to PTH in PHP leads to hypocalcemia. Hypocalcemia is hypothesized as one of the potential causes of disturbances of the tooth development, e.g., enamel opacities and enamel hypoplasia (Garfunkel et al., 1979; Gao et al., 2015). Studies on dental manifestations of the various types of Ns-HypoPT and PHP, e.g., enamel hypoplasia, enamel opacities, hypodontia, root deviations, and eruption disturbances are, however, often mentioned with only few references (Goswami et al., 2009; Velez et al., 2009; Kamarthi et al., 2013). Furthermore, dental findings are often reported as general characteristics of patients with Ns-HypoPT and PHP (Kamarthi et al., 2013).

A number of more recent and larger studies on 22q11DS describe in more details the dental findings in this specific subgroup (Klingberg et al., 2007; Nordgarden et al., 2012). As mentioned above, not all patients with 22q11DS have HypoPT. Thus, a full understanding of the dental features related to Ns-HypoPT and PHP diseases is not available.

The aim of the present study was to conduct a systematic review reporting on dental findings in patients with Ns-HypoPT and PHP.

## Materials and methods

The systematic review was based on PRISMA (Preferred Reporting Items for Systematic reviews and Meta-Analyses; Moher et al., 2009) and contained the PICO elements (Participants, intervention, comparison, outcome, and study design). In collaboration with an experienced research librarian (JLS), the first author conducted the search for relevant publications in four databases, PubMed, Web of Science, Scopus, and Embase.

### Search strategy

The literature search was based on the central terms in the classification of genetic and autoimmune causes of hypoparathyroidism (Table 1).

**Table 1:** Classification of genetic and autoimmune causes of hypoparathyroidism.

**Genetic**	**OMIM #**
PTH biosynthesis/secretion	
Familial isolated hypoparathyroidism, autosomal recessive (chromosome 6p24.2, *GCM2*)	146200
Hypoparathyroidism, autosomal recessive (chromosome 11p15.3, *PTH*)	146200
Hypoparathyroidism, autosomal dominant (chromosome 11p15.3, *PTH*)	146200
Calcium Sensing Receptor (*CaSR*)	
Autosomal Dominant Hypocalcemia, ADH (chromosom 13q13.3-q21.1, *CaSR*)	601198
Autosomal Dominant Hypocalcemia with Bartters syndrome, ADH (chromosom 13q13.3-q21.1, *CaSR*)	601198
Parathyroid development	
22q11 deletion (chromosome 22q11, *TBX1*)	188400
Hypoparathyroidism, retardation and dysmorphism syndrome, HRDS (chromosome 1q42.3)	241410
Sanjad Sakati Syndrome	
Kenney-Caffey syndrome 1 and 2. Arab descent	244460
Hypoparathyroidism, sensorineural deafness and renal disease, HDR (chromosome 10p14)	142655
Familial isolated hypoparathyroidism, autosomal recessive (chromosome 6p24.2, *GCM2*)	146200
Hypoparathyroidism, X-linked, HYPX (chromosome Xq26-q27)	307700
Mitochondrial disease	
Kears-Sayre syndrome, KSS	530000
Pearson Marrow-Pancreas Syndrome	557000
Mitochondrial myopathy, encephalopathy., lactic acidosis and stroke-like episodes, MELAS	540000
Mitochondrial trifunctional Protein Deficiency, MTPD	609015
Diabetes and Deafness, Maternally Inherited, MIDD	520000
Autoimmune	
Autoimmune Polyglandular Syndrome type 1, APS-1	240300
Autoimmune Polyendocrinopthy-candidiasis-esctodermal dystrophy, APECED (chromosome 21q22.3, *AIRE*)	607358
Anti-CaSR antibodies	

**Figure 1:**
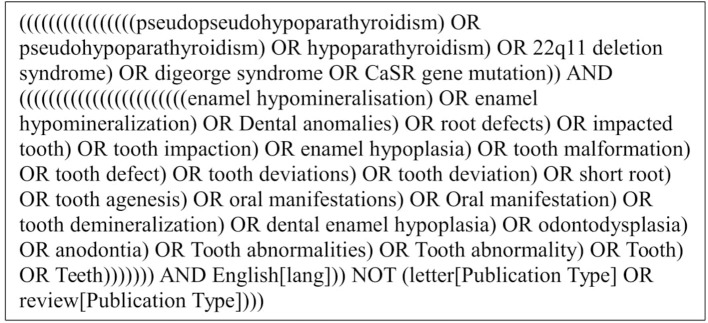
Search string used for the establishment of the study material.

Regarding PICOS, the population was defined through the following key words: “pseudopseudohypoparathyroidism,” “pseudohypoparathyroidism,” “hypoparathyroidism,” “22q11 deletion syndrome,” “DiGeorge syndrome,” and “CaSR gene mutation.” These words were connected with the Boolean operator “OR.” To determine the outcomes, the key words “Enamel hypomineralization,” “enamel hypomineralization,” “dental anomalies,” “root defects,” “impacted tooth,” “tooth impaction,” “enamel hypoplasia,” “tooth malformation,” “tooth defect,” “tooth deviation,” “tooth deviations,” “tooth deviation,” “tooth abnormalities,” “tooth abnormality,” “short root,” “tooth agenesis,” “oral manifestations,” “tooth demineralization,” “dental enamel hypoplasia,” “odontodysplasia,” “anodontia,” “tooth abnormalities,” “dental enamel,” “tooth,” and “teeth” were connected with the Boolean operator “OR.” Both the population and the outcome were connected with the Boolean operator “AND.” Interventions and comparators were not included in the search term due to low number of studies. The full electronic search strategy for PubMed is shown in Figure 1. In addition to the original search, a hand-search of the reference lists of the publications entered into the review was performed, with special focus on the known Scandinavian authors, e.g., Børglum, Klingberg, and Nordgarden (Børglum Jensen et al., 1983; Klingberg et al., 2005; Nordgarden et al., 2012). Lastly, an additional search on Scandinavian languages (Danish, Swedish, and Norwegian) was performed.

### Selection criteria and data extraction

**Figure 2:**
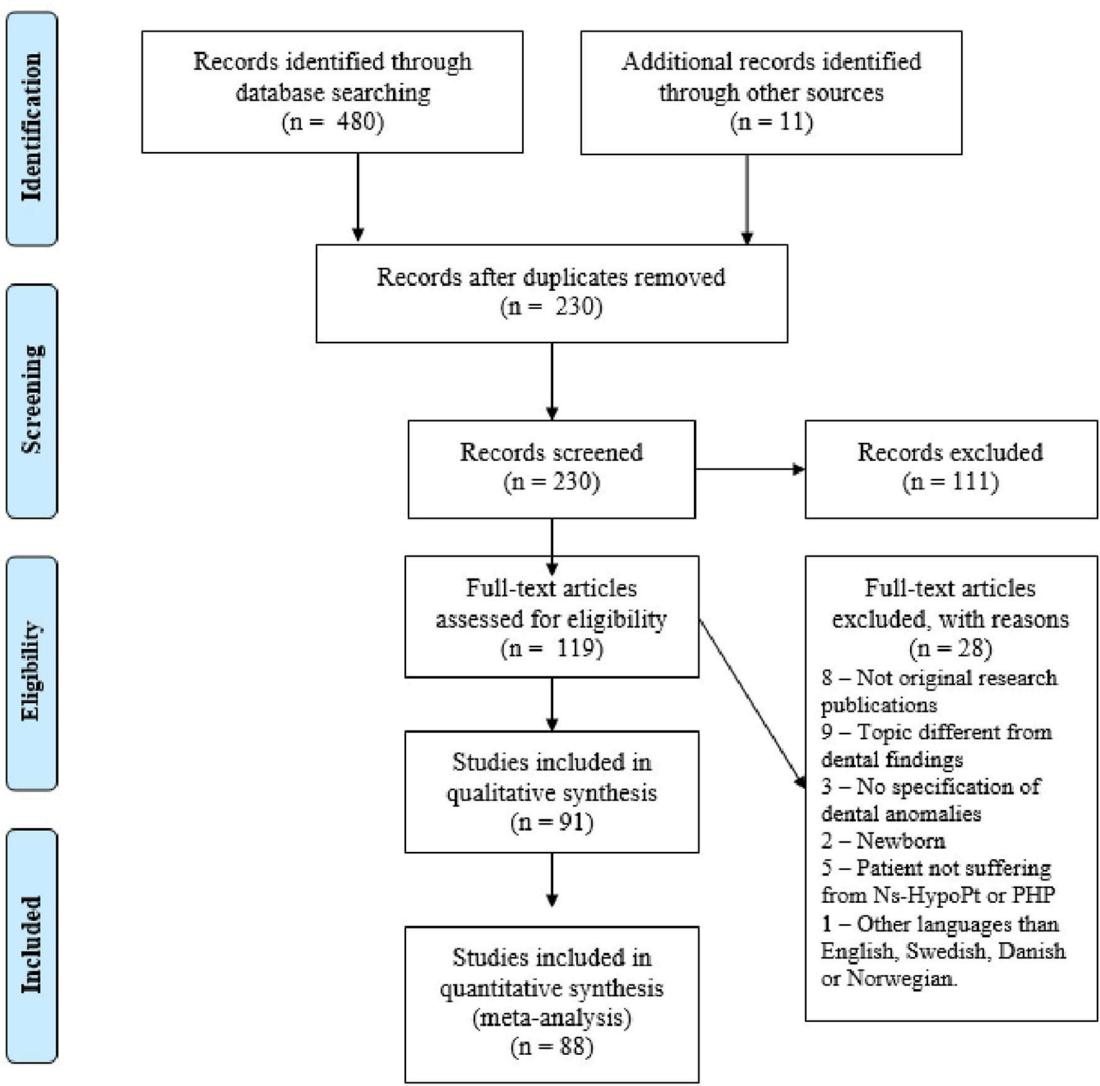
Flowchart illustrating the extraction of publications for the present review.

After identification of relevant publications, the duplicates were removed. The selection of publications was conducted in two phases, based on (1) the title and abstract, and (2) the full-text publication (Figure 2). The abstracts were included when they met the following inclusion criteria or when the first author was in doubt. All original reports, studying dental characteristics in Ns-HypoPT and PHP, were considered eligible for the present review. The systematic literature search included publications published in English up to date (January 2018). The exclusion criteria was as following: not original research publications (reviews, editorials, conference abstract, book chapter, letter, or commentaries), topic different from dental findings, animal studies, description of patients without teeth or newborn, publications without specification of dental anomalies, patients not suffering from Ns-HypoPT or PHP, and languages different from English, Danish, Swedish, or Norwegian.

The reasons for exclusion of publications are mentioned in Figure 2. The publications were inserted into the EndNote X8® software, and a list of references was generated for further analysis and selection.

### Quality assessment

We aimed to assess the publications according to Newcastle-Ottawa Quality Assessment Scale (Hospital, 2014) (NOS), Centre for Evidence-Based Medicine (CEBM) (Medicin, 2017), and Strength of Recommendation Taxonomy (SORT) (Ebell et al., 2004). Results were reported in five tables, two concerning Ns-HypoPT (Tables 2, 3), and another two related to PHP (Tables 4, 5) and one related to quality assessment (Table 6).

**Table 2:** Characteristics of studies on patients with non-surgical hypoparathyroidism.

**References**	**Design**	**Total no. of patients**	**Mean age (yr) [age range]**	**Gender M/F**	**Country of origin of patients**	**No. of patients with genetic analysis (genetic diagnose verified)**	**Biochemical test^a^ (no. of patients/total)**	**No. of patients with Hypo-PT^b^**
**A: IDIOPATHIC HYPOPARATHYROIDISM (NO. OF STUDIES IN SUBGROUPS** ***n*** = **25, TOTAL NO. OF PATIENTS IN SUBGROUP** ***n*** = **77)**								
Jensen et al., 1981	Case series	5	29 [12–67]	1/4	Denmark	0 (0)	1, 2, 3	5
Kelly et al., 2009	Case report	1	9	0/1	Brazil	0 (0)	1, 2, 3	1
Lyles et al., 1985	Case report	1	25	1/0	USA	0 (0)	1, 2	1
Moshkowitz et al., 1969	Case report	1	10	0/1	Israel	0 (0)	1, 2, 4	1
Assif, 1977	Case report	1	15.5	1/0	Israel	0 (0)	1, 2	–
Frensilli et al., 1971	Case series	3	26 [17–42]	2/1	USA	0 (0)	1, 2, 4	3
Kamarthi et al., 2013	Case report	1	22	1/0	India	0 (0)	1, 2, 3	1
Srirangarajan et al., 2014	Case report	1	40	0/1	India	0 (0)	1, 3	1
Lindeberg, 1979	Case report	1	16	0/1	Denmark	0 (0)	1, 2, 3	1
Lovestedt, 1971	Case report	1	14	0/1	USA	0 (0)	–	–
Illum et al., 1981	Case series	11*	41 [12–67]	4/7	Denmark	0 (0)	1, 2, 3 (3/11)	5
Harrell, 1983	Case report	1	27	1/0	England	0 (0)	1, 3	1
Nortjé, 2013	Case report	1	22	0/1	South Africa	0 (0)	1, 2	–
Pisanty, 1966	Case report	1	10	0/1	Persian	0 (0)	1, 2, 4	1
Riley, 1969	Case series	2	13 [13-13]	0/2	USA	0 (0)	1, 2, 4	2
Nikiforuk and Fraser, 1981	Case series	17	< 18	–	Canada	0 (0)	1, 2	17
Myllärniemi and Perheentupa, 1978	Case series	3	12 [2-22]	0/3	Finland	0 (0)	–	3
de Carvalho et al., 1986	Case report	1	45	1/0	Denmark	0 (0)	1, 2, 3	1
Hansted and Holst, 1952	Case report	1	19	0/1	Denmark	0 (0)	–	–
Humphreys, 1939	Case report	1	23	0/1	England	0 (0)	1, 4	1
Nally, 1970	Case report	1	13	0/1	England	0 (0)	1	–
Hinrichs, 1956	Case series	5	12 [6-18]	3/2	USA	0 (0)	1, 2, 4 (1/5)	1
Thew and Goulston, 1962	Case report	1	27	1/0	Australia	0 (0)	1,4	1
Mohsenipour et al., 2017	Case series	12	< 18	–	Iran	0 (0)	–	–
Sjöberg, 1966	Case series	3	22 [18–25]	1/2	Sweden	0 (0)	1, 2, 4	1
Subtotal		77						48
**B: CONGENITAL HYPOPARATHYROIDISM (NO. OF STUDIES IN SUBGROUPS** ***n*** = **2, TOTAL NO. OF PATIENTS IN SUBGROUP** ***n*** = **2)**								
Goepferd and Flaitz, 1981	Case report	1	14	0/1	USA	0 (0)	1, 2,4	1
Ingemarsson, 1984	Case report	1	6	0/1	Denmark	0 (0)	1, 2, 3	1
Subtotal		2						2
**C: 22q11 DELETION SYNDROME (NO. OF STUDIES IN SUBGROUPS** ***n*** = **16, TOTAL NO. OF PATIENTS IN SUBGROUP** ***n*** = **309)**								
Klingberg et al., 2007	Cross-sectional	29	12 [2–36]	13/16	Sweden	0 (0)	–	6
Lewyllie et al., 2017	Cross-sectional	20	9 [5–14]	12/8	Belgium	0 (0)	–	–
Toka et al., 2010	Case series	5	12 [6–15]	3/2	Germany	0 (0)	1 (1/5), 3 (1/5)	2
Heliövaara et al., 2011	Cross-sectional	45	8 [6–13]	22/23	Finland	45 (45)	–	–
Nordgarden et al., 2012	Cross-sectional	50	10 [2–44]	23/27	Norway	50 (50)	–	12
Matthews-Brzozowska et al., 2015	Case report	1	7	1/0	Poland	1 (1)	–	–
Børglum Jensen et al., 1983	Case report	1	8	0/1	Denmark	1 (1)	1, 2, 4	1
Fukui et al., 2000	Case series	2	13;13	2/0	Japan	2 (1)	1 (2/2), 4 (1/2)	2
Klingberg et al., 2002	Cross-sectional	53	11 [3–43]	23/30	Sweden	0 (0)	3 (9/53)	9
da Silva Dalben et al., 2008	Case series	26	18 [7–48]	11/15	Brazil	0 (0)	–	–
Oberoi et al., 2011	Cross-sectional	56	[3–14]	27/29	USA	0 (0)	–	–
Laccetta et al., 2015	Case report	1	5	1/0	Italy	1 (1)	–	–
Klingberg et al., 2005	Case series	15	11 [5-19]	–	Sweden	0 (0)	1 (7/15)	7
Yang et al., 2005	Case report	1	23	0/1	Taiwan	1 (1)	1, 2	***
Oberoi and Vargervik, 2005	Case series	3	9 [6–12]	1/2	USA	3 (3)	–	–
Jaquez et al., 1997	Case report	1	8	0/1	USA	1 (1)	–	–
Subtotal		309						39
**D: AUTOIMMUNE POLYGLANDULAR SYNDROME TYPE 1 (NO. OF STUDIES IN SUBGROUPS** ***n*** = **22, TOTAL NO. OF PATIENTS IN SUBGROUP** ***n*** = **233)**								
Greenberg et al., 1969	Case report	1	16	1/0	USA	0 (0)	1, 2, 4	1
Pisanty and Garfunkel, 1977	Case series	6	19 [10–30]	3/3	Israel/Persian	0 (0)	–	6
Hermans et al., 1969	Case series	3	27 [25–29]	1/2	USA	0 (0)	1 (1/3), 2 (1/3)	1
Kollios et al., 2011	Case report	1	11	0/1	Switzerland	1 (1)	1, 2	1
Perniola et al., 1998	Case series	4	15 [9–20]	2/2	Italy	0 (0)	–	3
Ahonen et al., 1990	Cross-sectional	68**	[1-60]	–	Finland	0 (0)	–	54
Bruserud et al., 2016	Cross-sectional	52	9 [0-43]	28/24	Norway	52 (52)	–	38
López-Jornet et al., 2005	Case report	1	10	0/1	Spain	0 (0)	1, 2, 3	1
Myllärniemi and Perheentupa, 1978	Case series	29	[4–28]	10/19	Finland	0 (0)	–	–
Lukinmaa et al., 1996	Case series	3	21 [17–26]	3/0	Finland	0 (0)	–	2
McGovern et al., 2008	Cross-sectional	16	13 [2–39]	7/9	Ireland	16 (16)	–	13
Ponranjini et al., 2012	Case report	1	35	0/1	India	0 (0)	1, 2, 3	1
Ferre et al., 2016	Prospective	35	20 [7–64]	14/21	North/South America	35 (35)	–	30
Bjanid et al., 2017	Case report	1	14	0/1	Poland	0 (0)	1, 2, 3	1
Porter et al., 1995	Case report	1	7	0/1	England	0 (0)	1, 2, 3	1
Walls and Soames, 1993	Case series	2	20 [19–21]	0/2	England	0 (0)	–	2
Porter et al., 1992	Case report	1	17	1/0	England	0 (0)	1, 3	–
Firth et al., 1997	Case report	1	21	0/1	Australia	0 (0)	–	1
Porter and Scully, 1986	Case series	2	30; −	1/1	England	0 (0)	–	1
Ali et al., 2014	Case report	1	15	1/0	Bangladesh	0 (0)	1, 3	1
Winer and Merke, 2000	Case report	1	10	1/0	USA	0 (0)	–	–
Pavlic and Waltimo-Siren, 2009	Case series	3	3 [9–15]	1/2	Slovenia	3 (3)	–	–
Subtotal		233						158
**E: SANJAD - SAKATI SYNDROME (NO. OF STUDIES IN SUBGROUPS** ***n*** = **4, TOTAL NO. OF PATIENTS IN SUBGROUP** ***n*** = **9)**								
Al-Malik, 2004	Case report	1	4	0/1	Saudi	0 (0)	1, 2, 3	1
Wasersprung et al., 2010	Case report	1	12	1/0	Bedouin origin	0 (0)	–	–
El Batawi, 2013	Case report	1	4	1/0	Saudi Arabia	0 (0)	1	–
Hershkovitz et al., 1995	Case series	6	2 [0–3]	3/3	Arab	0 (0)	1, 2, 3	6
Subtotal		9						7
Total		630 (603****)					254

–, *No data reported,*

* Only nine patients had oral examination preformed.

The remaining two patients had no teeth,

** Only 43 had oral examination,

*** Hypocalcemia, but normal PTH level 2,711 pg/ml,

**** *603 patients had oral examination performed*.

a *Biochemical tests carried out: 1: low calcium serum concentration, 2: high phosphate concentration, 3: low serum parathyroid hormone (PTH), 4: a rise in the plasma calcium concentration or/and a drop in plasma phosphate concentration in response to the injection of parathyroid hormone, 5: high PTH level, 6: PTH level and thyroid function test*.

b *Hypo-PT, hypoparathyroidism*.

**Table 3:** Reporting of oro-dental findings in patients with non-surgical hypoparathyroidism.

**References**	**Enamel opacity**	**Enamel hypoplasia**	**Crown size**	**Eruption disturbances^a^**	**Hypodontia**	**Root deviations^b^**	**Blunting of root apex**	**Pulp deviations^c^**	**SMCI^d^**
**A: IDIOPATHIC HYPOPARATHYROIDISM (NO. OF STUDIES IN SUBGROUPS** ***n** =* **25, TOTAL NO. OF PATIENTS IN SUBGROUP** ***n*** = **77)**									
Jensen et al., 1981	(1/5)	(1/5)	Reduced (1/5) normal (4/5)	1 (2/5), 2 (4/5)	(3/5)	(1/5)	(1/5)	–	–
Kelly et al., 2009	(1/1)	(1/1)	Reduced	1 (1/1)	–	(1/1)	(1/1)	–	–
Lyles et al., 1985	–	(1/1)	–	–	–	–	–	(1/1)	-
Moshkowitz et al., 1969	(1/1)	(1/1)	–	–	–	–	–	–	–
Assif, 1977	–	(1/1)	–	1 (1/1), 3 (1/1)	–	(1/1)	(1/1)	(1/1)	–
Frensilli et al., 1971	–	(3/3)	–	3 (1/3)	–	(3/3)	–	(1/3)	–
Kamarthi et al., 2013	–	(1/1)	Reduced (microdontia)	2 (1/1)	–	(1/1)	–	(1/1)	–
Srirangarajan et al., 2014	–	–	–	–	–	(1/1)	–	–	–
Lindeberg, 1979	–	–	Reduced (peg shaped)	–	(1/1)	–	–	–	–
Lovestedt, 1971	–	(1/1)**	–	–	–	–	–	–	–
Illum et al., 1981	–	(3/9)	–	1 (1/9), 3 (3/9)	(4/9)	(3/9)	–	–	–
Harrell, 1983	–	(1/1)	–	–	–	–	–	–	–
Nortjé, 2013	–	(1/1)	–	2 (1/1)	–	(1/1)	–	–	–
Pisanty, 1966	(1/1)	(1/1)	–	–	–	–	–	–	–
Riley, 1969	–	(2/2)	–	1 (1/2)	–	(1/2)	–	–	–
Nikiforuk and Fraser, 1981	–	(14/17)	–	–	–	–	–	(0/17)	–
Myllärniemi and Perheentupa, 1978	–	None	–	–	–	–	–	–	–
de Carvalho et al., 1986 ***	–	–	–	None	–	–	–	–	–
Hansted and Holst, 1952	–	(1/1)	–	3 (1/1)	(1/1)	-	-	(1/1)	–
Humphreys, 1939	–	(1/1)	–	–	–	–	–	–	–
Nally, 1970	–	(1/1)	–	–	(1/1)	–	–	–	–
Hinrichs, 1956	–	(3/5)	Normal (1/5)	1 (1/5), 2 (1/5), 3 (1/5)	(2/5)	(2/5)	–	(4/5)	–
Thew and Goulston, 1962	–	–	Reduced (microdontia)	–	(1/1)	(1/1)	–	(1/1)	–
Mohsenipour et al., 2017	–	(6/12)	–	1 (4/12)	–	–	–	(4/12)	–
Sjöberg, 1966	–	(2/3)	–	–	–	–	–	–	–
Subtotal	4	46		1 (11), 2 (7), 3 (7)	13	16	3	14	0
**B: CONGENITAL HYPOPARATHYROIDISM (NO. OF STUDIES IN SUBGROUPS** ***n*** = **2, TOTAL NO. OF PATIENTS IN SUBGROUP** ***n*** = **2)**									
Goepferd and Flaitz, 1981	–	(1/1)	–	1 (1/1)	–	(1/1)	–	–	–
Ingemarsson, 1984	(1/1)	(1/1)	–	1 (1/1)	*	–	–	–	–
Subtotal	1	2		1 (2)	0	1	0	0	0
**C: 22q11 DELETION SYNDROME (NO. OF STUDIES IN SUBGROUPS** ***n*** = **16, TOTAL NO. OF PATIENTS IN SUBGROUP** ***n*** = **309)**									
Klingberg et al., 2007	(11/29)	(8/29)	–	–	(5/29)	–	–	–	–
Lewyllie et al., 2017	–	(2/20)	–	2 (3/20)	(4/20)	–	–	–	–
Toka et al., 2010	(2/5)	(3/5)	Reduced (1/5)	1 (1/5)	–	–	–	–	–
Heliövaara et al., 2011	–	–	–	None	(8/45)	–	–	–	–
Nordgarden et al., 2012	(29/50)	(21/50)	–	–	(6/40)	–	–	–	–
Matthews-Brzozowska et al., 2015	–	–	–	1 (1/1)	none	–	–	–	–
Børglum Jensen et al., 1983	–	(1/1)	–	–	–	–	–	–	–
Fukui et al., 2000	(1/2)	(2/2)	–	1 (2/2)	(2/2)	–	–	–	–
Klingberg et al., 2002	(23/53)	(16/53)	Reduced (peg shaped) (8/53)	1 (9/53)	(7/53)	–	–	–	–
da Silva Dalben et al., 2008	(15/26)	(4/26)	Extra cusp (4/26) missing cusp (9/26) reduced (microdontia) (2/26)	–	(6/26)	–	–	–	–
Oberoi et al., 2011	–	–	–	1 (9/56)	(7/56)	–	–	–	–
Laccetta et al., 2015	–	(1/1)	–	–	–	–	–	–	–
Klingberg et al., 2005	(11/15)	(5/15)	–	–	–	–	–	–	–
Yang et al., 2005	–	–	–	–	–	–	–	–	(1/1)
Oberoi and Vargervik, 2005	(0/3)	(0/3)	Normal	None	(2/3)	None	None	None	(1/3)
Jaquez et al., 1997	–	–	–	–	–	–	–	–	–
Subtotal	92	63		1 (22), 2 (3)	53	0	0	0	2
**D: AUTOIMMUNE POLYGLANDULAR SYNDROME TYPE 1 (NO. OF STUDIES IN SUBGROUPS** ***n*** = **22, TOTAL NO. OF PATIENTS IN SUBGROUP** ***n*** = **233)**									
Greenberg et al., 1969	–	(1/1)	–	–	4	1	–	–	–
Pisanty and Garfunkel, 1977	–	(2/6)	–	2 (2/6)	(2/6)	(6/6)	–	(4/6)	-
Hermans et al., 1969	–	(1/3)	–	–	–	–	–	–	–
Kollios et al., 2011	–	(1/1)	–	–	–	–	–	–	–
Perniola et al., 1998	–	(4/4)	–	–	–	–	–	–	–
Ahonen et al., 1990	–	(33/43)	–	–	–	–	–	–	–
Bruserud et al., 2016	(5/31)	(18/31)	–	–	–	–	–	–	–
López-Jornet et al., 2005	–	(1/1)	–	–	–	–	–	–	–
Myllärniemi and Perheentupa, 1978	–	(23/29)	–	3 (3/29)	–	(0/29)	–	–	–
Lukinmaa et al., 1996	–	(3/3)	–	–	–	–	–	–	–
McGovern et al., 2008	(10/16)	(10/16)	–	–	–	–	–	–	–
Ponranjini et al., 2012	–	(1/1)	–	1 (1/1), 2 (1/1)	–	(1/1)	–	–	–
Ferre et al., 2016	–	(30/35)	–	–	–	–	–	–	–
Bjanid et al., 2017	–	(1/1)	–	–	–	–	–	–	–
Porter et al., 1995	–	(1/1)	–	–	–	–	–	–	–
Walls and Soames, 1993	(2/2)	(2/2)	–	3 (1/2)	–	–	–	–	–
Porter et al., 1992	–	(1/1)	–	–	–	–	–	–	–
Firth et al., 1997	–	(1/1)	–	–	–	–	–	–	–
Porter and Scully, 1986	–	(2/2)	–	–	–	–	–	–	–
Ali et al., 2014	–	(1/1)	–	–	–	–	–	–	–
Winer and Merke, 2000	–	(1/1)	–	–	–	–	–	–	–
Pavlic and Waltimo-Siren, 2009	(1/3)	(3/3)	–	1 (1/3)	–	–	–	–	–
Subtotal	17	139		1 (2), 2 (3), 3 (4)	6	8	0	4	0
**E: SANJAD - SAKATI SYNDROME (NO. OF STUDIES IN SUBGROUPS** ***n*** = **4, TOTAL NO. OF PATIENTS IN SUBGROUP** ***n*** = **9)**									
Al-Malik, 2004	–	(1/1)	Reduced (microdontia)	–	–	–	–	–	–
Wasersprung et al., 2010	–	-	Reduced (microdontia)	1 (1/1)	(1/1)	–	–	(1/1)	–
El Batawi, 2013	–	–	Reduced (microdontia)	–	–	–	–	–	–
Hershkovitz et al., 1995	–	–	–	1 (****/6)	–	–	–	–	–
Subtotal	0	1		1 (1+^****^)	1	0	0	1	0
Total	115	251		1 (38+^****^), 2 (13), 3 (11)	73	25	3	19	4

–*, No data reported*.

* *Too early to diagnose aplasia for the missing 15*.

** *>Pitting seen in clinical photos in the publication*.

*** *De Carvalho study only reported denture and no impacted teeth*.

**** *Reported as a common abnormality, however, did not report the exact no. of patient*.

a *Eruption disturbances: 1: delayed eruption, 2: impaction, 3: retention*.

b *Root deviations: Short root and/or thin root and/or incomplete root formation*.

c *Pulp deviations: Widened and/or calcification of pulp*.

d *SMCI, Single maxillary central incisor*.

**Table 4:** Characteristics of studies on patients with pseudohypoparathyroidism.

**References**	**Design**	**Total no. of patients**	**Mean age (yr) [age range]**	**Gender M/F**	**Country**	**Diagnose**	**No of patients with genetic analysis (genetic diagnose verified)**	**Biochemical tests^a^**
**PSEUDOHYPOPARATHYROIDISM (NO. OF STUDIES** ***n*** = **21, TOTAL NO. OF PATIENTS** ***n*** = **56)**								
Jensen et al., 1981	Case series	6	24 [10–47]	3/3	Denmark	PHP	0 (0)	1, 2, 3
Okano et al., 1969	Case report	1	22	1/0	Japan	PHP	0 (0)	6
Ritchie, 1965	Case series	4	19 [10–24]	1/3	England	PHP	0 (0)	–
Croft et al., 1965	Case report	1	12	0/1	Brazil	PHP	0 (0)	1, 2, 6
Cohen, 1991	Case report	1	21	1/0	USA	PHP	0 (0)	–
Illum et al., 1980	Case series	6	24 [10–47]	3/3	Denmark	PHP	0 (0)	1, 5
Witkop, 1976	Case report	1	12	0/1	–	PHP	0 (0)	–
Myllärniemi and Perheentupa, 1978	Case series	2	11 [9-14]	1/1	Finland	PHP	0 (0)	–
Nikiforuk and Fraser, 1981	Case series	4	< 18	–	Canada	PHP	0 (0)	1, 2
Lagarde et al., 1989	Case report	1	17	–	France	PHP	0 (0)	–
Glynne et al., 1972	Case report	1	32	0/1	Scotland	PHP	0 (0)	1, 6
Storm et al., 1985	Case report	1	3	0/1	Denmark	PPHP	0 (0)	–
Velez et al., 2009	Case report	1	14	0/1	African American	AHO	0 (0)	1
Gomes et al., 2002	Case report	1	17	0/1	Brazil	AHO	1 (1)	1, 2
Hugar et al., 2014	Case report	1	32	1/0	India	AHO	0 (0)	1, 6
Goswami et al., 2009	Case report	1	13	0/1	India	AHO	0 (0)	1, 2
Brown and Aaron, 1991	Case report	1	14	1/0	USA	AHO	0 (0)	1
Sengupta et al., 2012	Case report	1	16	0/1	India	AHO	0 (0)	1, 2, 5
Delantoni et al., 2017	Case report	1	28	1/0	Greece	Type 1a	0 (0)	1, 2
Reis et al., 2016	Case series	6	23 [8–32]	3/3	Brazil	AHO	6 (6)	–
Reis et al., 2016	Case series	13	29 [10–48]	7/6	Brazil	Type 2b	13 (13)	–
Gallacher et al., 2017	Case report	1	23	1/0	United Kingdom	Type 1b	1 (1)	1, 5
Total		56						

–*, No data reported*.

a *Biochemical test carried out: 1: low calcium serum concentration, 2: high phosphate concentration, 3: low serum parathyroid hormone (PTH), 4: a rise in the plasma calcium concentration or/and a drop in plasma phosphate concentration in response to the injection of parathyroid hormone, 5: high PTH level, 6: PTH level and thyroid function test*.

**Table 5:** Reporting of oro-dental findings in patients with pseudohypoparathyroidism.

**References**	**Enamel opacity**	**Enamel hypoplasia**	**Crown size**	**Eruption disturbances^a^**	**Hypodontia**	**Root deviations^b^**	**Blunting of root apex**	**Pulp deviations^c^**	**Lamina dura**
**PSEUDOHYPOPARATHYROIDISM (NO. OF STUDIES** ***n*** = **21, TOTAL NO. OF PATIENTS** ***n*** = **56)**									
Jensen et al., 1981	(1/6)	(5/6)	Normal	1 (3/6), 2 (3/6)	(4/6)	(2/6)	(4/6)	(1/6)	Thickening (1/6)
Okano et al., 1969	–	(1/1)	–	3 (1/1)	(1/1)	–	–	–	–
Ritchie, 1965	–	(3/4)	Reduced (2/4)	1 (1/4), 3 (1/4)	(2/4)	(2/4)	(1/4)	(3/4)	–
Croft et al., 1965	(0/1)	(1/1)	Normal	3 (1/1)	(1/1)	–	–	(1/1)	–
Cohen, 1991	–	(1/1)	–	–	–	(1/1)	(1/1)	–	–
Illum et al., 1980	–	(6/6)	-	1 (?*/6)	(?/6)	(?/6)	–	–	–
Witkop, 1976	–	(1/1)	Reduced (peg shaped)	1 (1/1)	(0/1)	(1/1)	–	(1/1)	–
Myllärniemi and Perheentupa, 1978	–	(0/2)	–	–	–	–	–	–	–
Nikiforuk and Fraser, 1981	–	(1/4)	–	–	–	–	–	(0/4)	–
Lagarde et al., 1989	–	(1/1)	Normal	–	(1/1)	(0/1)	–	(1/1)	–
Glynne et al., 1972	–	(1/1)	–	3 (1/1)	–	(1/1)	(1/1)	–	–
Storm et al., 1985	–	(1/1)	–	None	–	–	–	–	–
Velez et al., 2009	–	(1/1)	–	2 (1/1)	–	(1/1)	–	(1/1)	Thickening (1/1)
Gomes et al., 2002	–	(0/1)	–	None	–	(0/1)	–	–	–
Hugar et al., 2014	–	(1/1)	–	2 (1/1)	(1/1)	–	–	(1/1)	Loss of lamina dura
Goswami et al., 2009	–	(1/1)	–	1 (1/1)	–	–	–	–	–
Brown and Aaron, 1991	–	(1/1)	Reduced (microdontia)	1 (1/1)	(1/1)	–	–	(1/1)	–
Sengupta et al., 2012	–	(1/1)	–	–	(1/1)	–	–	–	–
Delantoni et al., 2017	–	(1/1)	–	2 (1/1)	–	(1/1)	(1/1)	–	–
Reis et al., 2016	–	(5/6)	–	3 (3/6)	none	(5/6)	(5/6)	(1/6)	–
Reis et al., 2016	–	(7/13)	–	3 (6/13)	(1/13)	(7/13)	(7/13)	(3/13)	–
Gallacher et al., 2017	–	(1/1)	Normal	3 (1/1)	(0/1)	(1/1)	(1/1)	(0/1)	(0/1)
Total	1	41		1 (8+?), 2 (5), 3 (14)	13+?	22+?	21	14	

–*, No data reported*.

* *Reported as a common abnormality, however, did not report the exact no. of patient*.

a *Eruption disturbances: 1: delayed eruption, 2: impaction, 3: retention*.

b *Pulp deviations: Widened and/or calcification of pulp*.

c *Root deviations: Short root and/or thin root and/or incomplete root formation*.

**Table 6:** Quality assessment using the Newcastle-Ottawa Scale.

**References**	**Year**	**Country**	**Criteria**									
				**Selection**				**Comparability**			**Outcome**			**Total score maximum**
			**Study design**	**1**	**2**	**3**	**4**	**5**	**6**	**7**	**8**	
Klingberg et al.	2007	Sweden	Cross-sectional			*		*	*					3
Lewyllie et al.	2017	Belgium	Cross-sectional			*			*					2
Heliövaara et al.	2011	Finland	Cross-sectional			*			*					2
Nordgarden et al.	2012	Norway	Cross-sectional			*			*					2
Klingberg et al.	2002	Sweden	Cross-sectional			*			*					2
Oberoi et al.	2011	USA	Cross-sectional			*			*					2
Ahonen et al.	1990	Finland	Cross-sectional	*		*			*					3
Bruserud et al.	2016	Norway	Cross-sectional			*			*					2
McGovern et al.	2008	Ireland	Cross-sectional			*		*	*					3
Ferre et al.	2016	North/South America	Prospective cohort			*		*	*					3

The use of

* *means that the publication qualified in the respective item of the Newcastle-Ottawa Scale. (1) Criteria. (2) Representativeness of the exposed cohort. (3) Selection of the non-exposed cohort. (4) Ascertainment of exposure. (5) Comparability on the basis of confounding control in the design or analysis. (6) Additional confounding control. (7) Assessment of outcome. (8) Duration of follow-up period*.

Data extraction was carried out using a tailored form that had been pilot-tested. The first author carried out the data extraction.

During the quality assessment, some of the findings were challenging, such as Fraser and co-workers publishing the same study in 1979 (Nikiforuk and Fraser, 1979), and in1981 (Nikiforuk and Fraser, 1981) and in 1982 (Fraser and Nikiforuk, 1982). Therefore, the findings in these three publications were included only once in the present review. The study by Garfunkel et al. (1979) was a histological assessment of teeth extracted from patients in the study by Pisanty et al. (Pisanty and Garfunkel, 1977). The reported findings were identical in both publications. Therefore, the findings were included only once. In addition, the reported treatment-related findings and soft tissue findings were not included, as these topics were not a part of the aim of the present review (Klingberg et al., 2002; Bruserud et al., 2016). Selective reporting and observational bias might be a risk, as case series and case reports were included, and this may affect the cumulative evidence.

## Results

### Study selection

The first author conducted the two selection phases based on the abstracts and the full-text publications. If difficulties in the interpretation of the information in the publications appeared, consensus was reached between the first author and two other authors (HG and DH). The results of the data extraction are displayed in Tables 2–5.

After having performed a systematic literature search, it became clear that the number, character, and quality of the publications on the subject were at a relatively low scientific level to provide a basis for making a systematic review according to the guidelines in NOS, CEMB, and SORT. High quality studies were almost absent, as most of the studies were case reports and case series (Tables 2–5). However, nine studies on Ns-HypoPT were cross-sectional studies, and one on Ns-HypoPt was a prospective cohort study. Using NOS for cohort studies, none of these studies were classified as high quality (Table 6). None of the studies concerning PHP met the inclusion criteria for NOS assessment. All studies were included in the review due to low number of quality studies. The reported diagnoses in the publications included in the present study were categorized according to the classification given in Table 1.

Tables 2–5 display the extracted results. Table 2 illustrates study characteristics for Ns-HypoPT studies (Humphreys, 1939; Hansted and Holst, 1952; Hinrichs, 1956; Thew and Goulston, 1962; Pisanty, 1966; Sjöberg, 1966; Greenberg et al., 1969; Hermans et al., 1969; Moshkowitz et al., 1969; Riley, 1969; Nally, 1970; Frensilli et al., 1971; Lovestedt, 1971; Assif, 1977; Pisanty and Garfunkel, 1977; Myllärniemi and Perheentupa, 1978; Lindeberg, 1979; Goepferd and Flaitz, 1981; Illum et al., 1981; Jensen et al., 1981; Nikiforuk and Fraser, 1981; Børglum Jensen et al., 1983; Harrell, 1983; Ingemarsson, 1984; Lyles et al., 1985; de Carvalho et al., 1986; Porter and Scully, 1986; Ahonen et al., 1990; Porter et al., 1992, 1995; Walls and Soames, 1993; Hershkovitz et al., 1995; Lukinmaa et al., 1996; Firth et al., 1997; Jaquez et al., 1997; Perniola et al., 1998; Fukui et al., 2000; Winer and Merke, 2000; Klingberg et al., 2002, 2005, 2007; Al-Malik, 2004; López-Jornet et al., 2005; Oberoi and Vargervik, 2005; Yang et al., 2005; da Silva Dalben et al., 2008; McGovern et al., 2008; Kelly et al., 2009; Pavlic and Waltimo-Siren, 2009; Toka et al., 2010; Wasersprung et al., 2010; Heliövaara et al., 2011; Kollios et al., 2011; Oberoi et al., 2011; Nordgarden et al., 2012; Ponranjini et al., 2012; El Batawi, 2013; Kamarthi et al., 2013; Nortjé, 2013; Ali et al., 2014; Srirangarajan et al., 2014; Laccetta et al., 2015; Matthews-Brzozowska et al., 2015; Bruserud et al., 2016; Ferre et al., 2016; Bjanid et al., 2017; Lewyllie et al., 2017; Mohsenipour et al., 2017) with subgroups A–E corresponding to the classification given in Table 1. Table 3 illustrates the oro-dental findings in Ns-HypoPT studies. Two studies (Jackson and Whyte, 1967; Kinirons and Glasgow, 1985) were not included in the tables, as they only reported on dentinal changes seen in light microscope. Table 4 illustrates study characteristics for all PHP studies (Croft et al., 1965; Ritchie, 1965; Okano et al., 1969; Glynne et al., 1972; Witkop, 1976; Myllärniemi and Perheentupa, 1978; Illum et al., 1980; Jensen et al., 1981; Nikiforuk and Fraser, 1981; Storm et al., 1985; Lagarde et al., 1989; Brown and Aaron, 1991; Cohen, 1991; Gomes et al., 2002; Goswami et al., 2009; Velez et al., 2009; Sengupta et al., 2012; Hugar et al., 2014; Reis et al., 2016; Delantoni et al., 2017; Gallacher et al., 2017) as a combined group, and Table 5 illustrates the oro-dental findings in PHP studies. The studies included were published between 1939 and 2017 (Humphreys, 1939; Lewyllie et al., 2017). The majority were case reports (52 papers) or case series (26 papers), and only nine cross-sectional and one prospective cohort study were included.

### Population characteristics

Only one of the included studies claimed the group of participants to be representative for the population studied (Ahonen et al., 1990). The number of participants with oral examination among the 88 studies included, ranged from 1 to 68 (Ahonen et al., 1990; Firth et al., 1997). The age of participants in the studies varied from 0 to 67 years. The total number of studies describing Ns-HypoPT were 68, comprising 630 patients in the five different subgroups A–E (Table 2). However, only 39 of the 309 patients in subgroup C (22q11DS) also reported having HypoPT, and 208 of the 233 patients in subgroup D (APS 1) underwent an oral examination. Out of 233 patients, only 154 had HypoPT. The total number of patients with Ns-HypoPT, who underwent an oral examination, was 603. The total number of studies describing PHP was 21, including a total of 56 patients (Table 4).

### Diagnostic criteria used in the publications

The studies lacked the use of international standardized criteria for the description of dental anomalies. Only few studies (*n* = 4) mentioned the diagnostic criteria used to assess dental anomalies, e.g., as dental opacities and enamel hypoplasia (Klingberg et al., 2002, 2007; da Silva Dalben et al., 2008; Nordgarden et al., 2012). In addition, no studies described the criteria for the distinction between impactions and retentions.

### Findings of dental characteristics

In 603 patients with Ns-HypoPT and oral examination, the most prevalent findings were dental tissue alterations as enamel hypoplasia [*n* = 251 (42%)], enamel opacities [*n* = 115 (19%)], quantitative dental anomaly as hypodontia [*n* = 73 (12%)], and eruption disturbances, especially delayed eruption [*n* > 38 (>6%) the exact number was not specified in all the studies; Table 3]. However, only 14 of the 68 studies (21%) on Ns-HypoPT reported information on the genetic molecular diagnosis of the disease. In the studies mentioned in Table 3, a number of additional findings as cemental hyperplasia, widened periodontal ligament space, widened apical foramen, thickening of lamina dura, hyperdontia, resorptions, forced bite, as well as osteitis with normal root formation were occasionally reported (Thew and Goulston, 1962; Frensilli et al., 1971; Jensen et al., 1981; Børglum Jensen et al., 1983; Toka et al., 2010; Nordgarden et al., 2012; El Batawi, 2013; Srirangarajan et al., 2014; Lewyllie et al., 2017). Dentinal changes were described in three publications (Jackson and Whyte, 1967; Kinirons and Glasgow, 1985; Fukui et al., 2000). Kinirons and Glasgow found no gross dentinal changes in three patients. In contrast, Jackson and Whyte and Fukui and co-workers found irregularities of calcification and matrix formation in one patient, respectively.

In patients with PHP (*n* = 56), the most frequently reported findings were dental tissue alterations as enamel hypoplasia [*n* = 41 (73%)], deviation of root morphology [*n*>22 (>39%)], blunting of root apex [*n* = 21 (38%)], and eruption disturbances, especially retention [*n* = 14 (25%); Table 5]. Only three studies reported detailed information on genetic molecular diagnosis. In addition to findings listed in Table 5, enamel pearls, widened apical foramen, as well as ankylosed teeth were occasionally reported in a limited number of studies (Croft et al., 1965; Jensen et al., 1981; Lagarde et al., 1989; Brown and Aaron, 1991).

## Discussion

Calcium and phosphate metabolism is affected in Ns-HypoPT and PHP and considered of importance to tooth development (Pindborg, 1982). Many different, but sporadically-occurring, dental features were found in patients with Ns-HypoPT and PHP. The most frequently reported dental findings in patients suffering from Ns-HypoPT were enamel hypoplasia, enamel opacities, hypodontia, and various types of eruption disturbances. The most frequently reported dental findings in patients with PHP were enamel hypoplasia, eruption disturbances, and deviation of root morphology. However, the character of studies and the number of cases, providing the basis of the studies, were too small to determine relatively, which dental anomalies were having the strongest association with the two diseases.

As in the dental field, well-defined diagnostic criteria also appear to be a challenge in the medical field. IHP, a condition with unknown etiology, is classified as a subgroup of Ns-HypoPT (Table 2). However, we cannot exclude the possibility that a few (*n* ≤ 15) of these patients with IHP might suffer from PHP. Description of verification of Hypo-PT was lacking and the details of the medical examinations underlying the diagnosis was not fully documented in some of these publications (Lovestedt, 1971; Assif, 1977; Illum et al., 1981; Nortjé, 2013; Mohsenipour et al., 2017). The majority of the case reports are published before 1985, and molecular genetic tests were not included in these papers. One study described a patient with acute meningo-encephalitis and, therefore, they made no attempt to establish whether it was a case of PHP or IHP (Assif, 1977), but the patient was diagnosed as IHP. Nine studies out of 25 studies reporting IHP (36%) performed a test (administration of parathyroid hormone) to rule out the diagnosis of PHP (Humphreys, 1939; Hinrichs, 1956; Thew and Goulston, 1962; Pisanty, 1966; Sjöberg, 1966; Moshkowitz et al., 1969; Riley, 1969; Frensilli et al., 1971; Illum et al., 1981). Only eight out of 25 studies (32%) provided information on PTH levels (Table 4), as an indication of these patients having Ns-HypoPT, most likely 22q11DS or APS 1. Some studies of IHP reported pulp deviations and root deviations (Assif, 1977; Illum et al., 1981) with a greater frequency than reported in the other subgroups of Ns-HypoPT. From a dental point of view, this might indicate that a few patients in the IHP group instead may have suffered from PHP. Furthermore, other studies on IHP reported on enamel opacities. This dental finding, however, is only reported few times in PHP patients, but occurs with a higher frequency in Ns-HypoPT, subgroups like 22q11DS and APS 1 (Pisanty, 1966; Moshkowitz et al., 1969; Jensen et al., 1981; Kelly et al., 2009). This could indicate that some of the patients denominated as having IHP in reality could have suffered from 22q11DS or APS 1.

Teeth develop in a predictable sequence over 20 years, and formation and/or eruption of teeth can be used to estimate age up to early adulthood (AlQahtani et al., 2014). This means that disturbances in processes related to tooth development at a particular time point can cause defects (e.g., enamel opacities or enamel hypoplasia) on the tooth at different localizations. Standardized terminology and criteria for diagnosis of enamel hypoplasia and enamel opacities was not used in the majority of the included studies in the present review. In addition, there was no distinction between retention and impaction, as these words seemed to be used synonymously (Illum et al., 1981; Jensen et al., 1981; Ponranjini et al., 2012; Delantoni et al., 2017). Furthermore, standard protocol was lacking for the overall oral examination in the majority of the publications. Selective reporting and observational bias might be a risk, as case series and case reports were included.

Inaccuracies in the terminology on enamel abnormalities were noted. Enamel anomalies described as white stainings (Goswami et al., 2009), chalky white areas (Lagarde et al., 1989), or dull white in color (Ritchie, 1965) are not the characteristics of opacities only. Dental fluorosis and caries can also cause white spot lesions, which were not discussed in the publications (Ritchie, 1965; Lagarde et al., 1989; Goswami et al., 2009). In addition, negative findings were not stated in a number of studies. Hence, it was unknown if negative findings were assessed or not. Furthermore, in studies with 22q11 DS or APS 1, it was not always possible to differentiate patients diagnosed with HypoPT from those without the diagnosis. It is therefore uncertain which of the reported dental findings are in fact associated with HypoPT.

Dental findings related to Ns-HypoPT and PHP differed; e.g., blunting of root apex was reported more frequently in patients with PHP than in patients with Ns-HypoPT. PHP is often diagnosed later in life, because only type 1a has a clear phenotype, and patients often develop hypocalcemia later than patients with Ns-HypoPT do. However, it has so far not been possible to establish any correlation between enamel hypoplasia and the age of diagnosis/treatment (Reis et al., 2016). Patients with PHP may be exposed to hypocalcemia later in life than other relevant patient categories. Therefore, this could explain why the root is affected more frequently in patients with PHP compared to patients with Ns-HypoPT.

Hypocalcemia is proposed to be involved in the etiology of enamel opacities and enamel hypoplasia. Studies have shown that enamel opacities and enamel hypoplasia develop in children who suffer from hypocalcemia during the period of enamel formation (secretion, mineralization, and maturation; Stimmler et al., 1973; Nikiforuk and Fraser, 1981; Fraser and Nikiforuk, 1982; Klingberg et al., 2005; Kelly et al., 2009). However, enamel opacities and hypoplasia are reported more often in the permanent than in the primary dentition (Nordgarden et al., 2012). Stimmler et al. (1973) found that enamel defects occurred in postnatally developed teeth of children who suffered from neonatal tetany caused by hypocalcemia. Nikiforuk and Fraser (1981) support this finding. They found that all patients with enamel hypoplasia had hypocalcemia. Moreover, they found no correlation between enamel hypoplasia and plasma phosphate concentration. They hypothesized that enamel hypoplasia is a more sensitive indicator of hypocalcaemia than the classic neurological indices (i.e., tetany and convulsion). In addition, an animal study showed that rats with thyro-parathyrodectomi-induced hypocalcemia developed incisors with enamel hypoplasia (Chardi et al., 1998). However, normal tooth formation may not only depend on calcium metabolism. In mice, *GCM2* and *PTH*, which are genes associated with Ns-HypoPT and PHP, are not expressed during cap stage of tooth germs formation. In contrast, *CaSR* and *AIRE* are moderately expressed (higher in lower molars than in upper molars) and *TBX1, GNAS* and *STX16* are highly expressed during tooth germs formation in mouse (Laugel-Haushalter et al., 2013). A direct effect during tooth development in humans mediated via *Tbx1, GNAS, STX16, CASR*, and *AIRE* is therefore possible. The etiology of enamel defects in patients suffering from APS 1 remains unclear (Perniola et al., 1998). Porter et al. (1995) reported an association of enamel defects with HypoPT, but Ahonen et al. (1990) suggested that there was no such association.

PTH receptor 1, *PTHR1*, a G protein-coupled receptor for parathyroid hormone and parathyroid hormone-like mutations, has shown to cause primary failure of tooth eruption (Decker et al., 2008). Failure of tooth eruption is also seen in Ns-HypoPT and patients with PHP. Primary failure of eruption is characterized by posterior rather than anterior tooth involvement; however, this is not the pattern seen in Ns-HypoPT and PHP. The etiology of failure of eruption in Ns-HypoPT and PHP must, therefore, be hypothesized to be different from that related to *PTHR1* mutations.

Worldwide interest in the association between Ns-HypoPT/PHP and dental findings is seen, as a substantial amount of the studies were performed in Asia, North and South America, and Europe. Most studies included both clinical and radiographic dental findings, which could be considered a strength. However, studies used different methods for the description of the patients, e.g., one study concerning IHP and one concerning PHP described dental findings based on only few X-rays (Lovestedt, 1971; Witkop, 1976), and a study on 22q11DS described dental findings based only on 38 exfoliated teeth from 15 different patients (Klingberg et al., 2005). Consequently, these studies did not include findings of teeth other than those that had been exfoliated or examined by radiographic examination. Hence, it is difficult to draw firm conclusions and to provide a full overview of the topic based on the available publications at present time.

In conclusion, the findings of the present review indicate a high prevalence of enamel hypoplasia and enamel opacities in patients suffering from Ns-HypoPT, and a high prevalence of enamel hypoplasia and root deviation in patients with PHP. None of the studies included in the present review were classified as high-quality studies; therefore, the results have to be interpreted cautiously. This confirms the need for further well-designed studies, for example, larger multicenter studies with identical shared protocol and definition of the diagnostic criteria as a groundwork for an identical and systematic collection of dental findings in patients with rare diseases as Ns-HypoPT and PHP.

## Author contributions

DH developed the idea of the manuscript. All authors contributed to design. JH established the literature search string with help from DH, HG. JH extracted data from publications. JH, LU, HG, and DH contributed to analysis and interpretation of data. JH wrote the first draft of the manuscript. All authors contributed to manuscript revision, read and approved the submitted version.

### Conflict of interest statement

The authors declare that the research was conducted in the absence of any commercial or financial relationships that could be construed as a potential conflict of interest. The reviewer ELN and handling Editor declared their shared affiliation.
